# Use of critical care resources during the first 2 weeks (February 24–March 8, 2020) of the Covid-19 outbreak in Italy

**DOI:** 10.1186/s13613-020-00750-z

**Published:** 2020-10-12

**Authors:** Tommaso Tonetti, Giacomo Grasselli, Alberto Zanella, Giacinto Pizzilli, Roberto Fumagalli, Simone Piva, Luca Lorini, Giorgio Iotti, Giuseppe Foti, Sergio Colombo, Luigi Vivona, Sandra Rossi, Massimo Girardis, Vanni Agnoletti, Anselmo Campagna, Giovanni Gordini, Paolo Navalesi, Annalisa Boscolo, Alessandro Graziano, Ilaria Valeri, Andrea Vianello, Danilo Cereda, Claudia Filippini, Maurizio Cecconi, Franco Locatelli, Michele Bartoletti, Maddalena Giannella, Pierluigi Viale, Massimo Antonelli, Stefano Nava, Antonio Pesenti, V. Marco Ranieri

**Affiliations:** 1 Alma Mater Studiorum, Dipartimento Di Scienze Mediche E Chirurgiche, Anesthesia and Intensive Care Medicine, Università Di Bologna, Policlinico Di Sant’Orsola, Via Massarenti, 9 40138 Bologna, Italy; 2 Fondazione IRCCS Ca’ Granda Ospedale Maggiore Policlinico, University of Milan, Milan, Italy; 3 Department of Pathophysiology and Transplantation, University of Milan, Milan, Italy; 4 Anesthesia and Critical Care, ASST Grande Ospedale Metropolitano Niguarda, University of Milano-Bicocca, Milan, Italy; 5 Anesthesia and Critical Care, ASST Spedali Civili, University of Brescia, Brescia, Italy; 6 Anesthesia and Critical Care, ASST Papa Giovanni XXIII, Bergamo, Italy; 7 Anesthesia and Critical Care, Fondazione IRCCS Policlinico San Matteo, Pavia, Italy; 8 Anesthesia and Critical Care, ASST Ospedale San Gerardo Di Monza, University of Milano-Bicocca, Milan, Italy; 9 Anesthesia and Critical Care, IRCCS San Raffaele Scientific Institute, Università Vita-Salute San Raffaele, Milan, Italy; 10 Anesthesia and Critical Care, Azienda Ospedaliero-Universitaria Di Parma, Parma, Italy; 11 Anesthesia and Critical Care, Policlinico Di Modena, Università Di Modena E Reggio Emilia, Modena, Italy; 12 Anesthesia and Critical Care Ospedale “M. Bufalini”, Cesena, Italy; 13 Assessorato Cura Della Persona, Regione Emilia-Romagna, Salute e Walfare, Bologna, Italy; 14 Anesthesia and Critical Care Ospedale, Ospedale Maggiore, Bologna, Italy; 15 Anesthesia and Critical Care, Department of Medicine, DIMED - University of Padua, University Hospital of Padua, Padua, Italy; 16 Respiratory Pathophysiology Division University-City Hospital of Padua, Padua, Italy; 17 Direzione Generale Welfare, Lombardy Region, Milan, Italy; 18 Dipartimento Di Scienze Chirurgiche, Università Di Torino, Torino, Italy; 19 Department of Anesthesia and Intensive Care, Humanitas Clinical and Research Center, Humanitas University, Milan, Italy; 20 Department of Biomedical Sciences, Pieve Emanuele, Milan, Italy; 21 Department of Pediatric Hematology and Oncology, Sapienza University of Rome, IRCCS Ospedale Pediatrico Bambino Gesù. President of the “Consiglio Superiore Di Sanità”, Rome, Italy; 22 Alma Mater Studiorum, Dipartimento Di Scienze Mediche E Chirurgiche, Infectious Diseases Unit, Università Di Bologna, Sant’Orsola-Malpighi Hospital, University of Bologna, Bologna, Italy; 23 Dept. of Intensive Care Emergency Medicine and Anesthesia, Fondazione Policlinico Universitario A. Gemelli IRCCS, Università Cattolica del Sacro Cuore, Rome, Italy; 24 Department of Clinical, Integrated, and Experimental Medicine (DIMES), Respiratory and Critical Care, Sant’Orsola Malpighi Hospital, Bologna, Italy

**Keywords:** COVID-19, Acute respiratory failure, ICU, Non-invasive ventilation, Rationing

## Abstract

**Background:**

A Covid-19 outbreak developed in Lombardy, Veneto and Emilia-Romagna (Italy) at the end of February 2020. Fear of an imminent saturation of available ICU beds generated the notion that rationing of intensive care resources could have been necessary.

**Results:**

In order to evaluate the impact of Covid-19 on the ICU capacity to manage critically ill patients, we performed a retrospective analysis of the first 2 weeks of the outbreak (February 24–March 8). Data were collected from regional registries and from a case report form sent to participating sites. ICU beds increased from 1545 to 1989 (28.7%), and patients receiving respiratory support outside the ICU increased from 4 (0.6%) to 260 (37.0%). Patients receiving respiratory support outside the ICU were significantly older [65 vs. 77 years], had more cerebrovascular (5.8 vs. 13.1%) and renal (5.3 vs. 10.0%) comorbidities and less obesity (31.4 vs. 15.5%) than patients admitted to the ICU. PaO_2_/FiO_2_ ratio, respiratory rate and arterial pH were higher [165 vs. 244; 20 vs. 24 breath/min; 7.40 vs. 7.46] and PaCO_2_ and base excess were lower [34 vs. 42 mmHg; 0.60 vs. 1.30] in patients receiving respiratory support outside the ICU than in patients admitted to the ICU, respectively.

**Conclusions:**

Increase in ICU beds and use of out-of-ICU respiratory support allowed effective management of the first 14 days of the Covid-19 outbreak, avoiding resource rationing.

## Introduction

Data regarding the impact of Covid-19 outbreak on the capacity of the health-care system to accomplish the need for ICU care are limited. The estimated need for intensive care unit (ICU) admission is variable, ranging between 5.0 [1], 7.0 [2] and 26.1% [3]. Reported ICU mortality ranges between 4 [3], 26 [4] 61 [2], and 67% [5]. This extreme variability has been attributed to differences in terms of beds availability, staff and organization of intensive care units [6].

On Thursday, 20th February 2020, the first cases of positivity for SARS-CoV-2 were recorded in Lombardy region, northern Italy. Since then, the number of patients with Corona Virus Disease-19 (Covid-19) and acute hypoxemic respiratory failure in three regions of northern Italy (Lombardy, Veneto and Emilia-Romagna) dramatically increased, subsequently leading to the call of a national emergency status [7].

A mathematical model of the occupation of intensive care resources in Italy predicted the saturation of the theoretical availability of beds on the national territory by mid-April 2020 [8]. In order to respond to such predicted growing need for ICU resources, on March 1st the Italian government published a notice, ordering to increase the number of ICU beds (https://www.salute.gov.it/portale/homeMobile.jsp) and approved a law decree that allocated 845 million euros to the public health service to bring the number of ICU beds for invasive mechanical ventilation to the 14% of the total hospital beds (https://www.gazzettaufficiale.it/eli/id/2020/03/09/20G00030/sg).

Since the spread of the SARS-CoV-2 virus is growing and critical care resources of public health systems are dramatically challenged [9], we reasoned that a better understanding of clinical management and ICU requirements for patients with severe Covid-19 at the very beginning of the outbreak may support resources planning and may help to set effective organizational and clinical interventions for the most seriously affected patients. The objective of the study was therefore to (1) describe the process of expansion of the ICU capacity in response to the Covid-19 outbreak during the first 2 weeks of the pandemics; (2) describe settings and modalities of care of acutely ill Covid-19 patients; (3) compare outcomes between critically ill patients with Covid-19 receiving care in or outside the ICU.

## Methods

We retrospectively studied consecutive critically ill patients with confirmed Covid-19 who were referred to the hospitals of the Lombardy, Veneto and Emilia-Romagna regions during the first 2 weeks of the Italian outbreak (February 24–March 8, 2020).

A confirmed case of Covid-19 was defined as a patient with a positive result on high-throughput sequencing or real-time reverse transcriptase-polymerase chain reaction assay of nasal and pharyngeal swab specimens [10].

In total, 30 hospitals (14 in Lombardy, 9 in Emilia-Romagna and 7 in Veneto) participated in the study. Institutional Review Boards reviewed the protocol and authorized data collection.

### Data collection

Data on ICU beds expansion and on total hospital and ICU admissions were gathered from registries of the regional ICUs coordinators of Lombardy (AP), Veneto (PN) and Emilia-Romagna (VMR) [7].

Moreover, a data collection form was circulated among participating ICUs and de-identified data on patients admitted in the ICU and receiving respiratory support outside the ICU were recorded 24 h after admission. In particular, demographics, comorbidities and basic physiological data were collected.

### System-wide changes to ICU and hospital capacity

In the initial 14 days of the epidemics in Northern Italy, ICU beds and personnel were made available by closing elective surgical admissions and centralizing to a limited number of single non-Covid-19 hub hospitals all neuro- and cardiac-surgical admissions. Moreover, ordinary availability of ICU beds in the three regions was increased from 1545 to 1989 (28.7%); in particular, ICU capacity increased by 41.4% (from 725 to 1025), 28.1% (from 370 to 474) and 8.9% (from 450 to 490) in Lombardy, Emilia-Romagna and Veneto, respectively. This was achieved by converting operating rooms, coronary units, step-down units and recovery rooms to fully equipped Covid-19 ICUs. Furthermore, the use of out-of-ICU respiratory support in the form of CPAP or NIV [11–13] was extended to many different wards, although initial reports suggested caution in the use of non-invasive respiratory support in Covid-19 patients due to the risk of transmission of infection [14].

### Clinical care

All patients included in the study underwent evaluation by a senior intensivist, who decided according to her/his clinical judgment and to local protocols whether to treat the patient in a ward under supervision of the ICU team or to admit the patient to the ICU. The criteria for ICU admission were: (a) failure of noninvasive respiratory support, defined as persistent hypoxemia, tachypnea and respiratory distress or development of hypercapnia despite the application of CPAP/NIV; (b) expected imminent need for invasive mechanical ventilation; (c) absence of a do-not-intubate order, as discussed collegially by the intensivist and the ward staff physicians caring for the patient.

At all sites out-of-ICU respiratory support was provided by care teams that included at least (i) a senior clinical staff with certified experience in intensive care medicine available around the clock; (ii) nurse support provided with a nurse/patients ratio ranging from 1:4 to 1:6; (iii) continuous monitoring of electrocardiogram trace, non-invasive blood pressure, oxygen saturation, and respiratory rate.

Conventional oxygen therapy was referred as applied through Venturi or no-rebreathing masks. Helmets were the interface systematically used to deliver CPAP. NIV was equally delivered through mask and helmets. High-flow oxygen therapy was adopted in some units as an alternative to CPAP.

Classification into oxygen therapy and non-invasive respiratory support followed the rule of the highest degree of support; accordingly, a patient receiving oxygen therapy at first and then escalating to non-invasive support was classified as receiving non-invasive support.

### Statistical analysis

Continuous variables were expressed as medians and interquartile ranges (IQR). Categorical variables were summarized as counts and percentages. No imputation was made for missing data. Statistical analyses were descriptive. Comparisons between groups were made using Wilcoxon rank-sum and Pearson’s Chi-square. All tests were 2-tailed and were considered significant if *p* < 0.05.

Twenty-eight-day mortality of patients admitted in the ICU through the period February 24–March 8, 2020 and of patients receiving respiratory support outside the ICU through the same period was evaluated using the method of Kaplan–Meier. Cumulative incidence of patients extubated and disconnected from mechanical ventilation was calculated and death was considered a competing event. Patients were followed up until April 5th.

All the analyses were performed with the use of SAS software, version 9.4 (SAS Institute Inc., Cary, NC).

## Results

In the period February 24th–March 8th, registries of the coordinating centers of Lombardy, Emilia-Romagna and Veneto showed that a total of 6378 patients were hospitalized for Covid-19 and a total of 805 were admitted and treated in the ICU (12.6%).

**Fig. 1:**
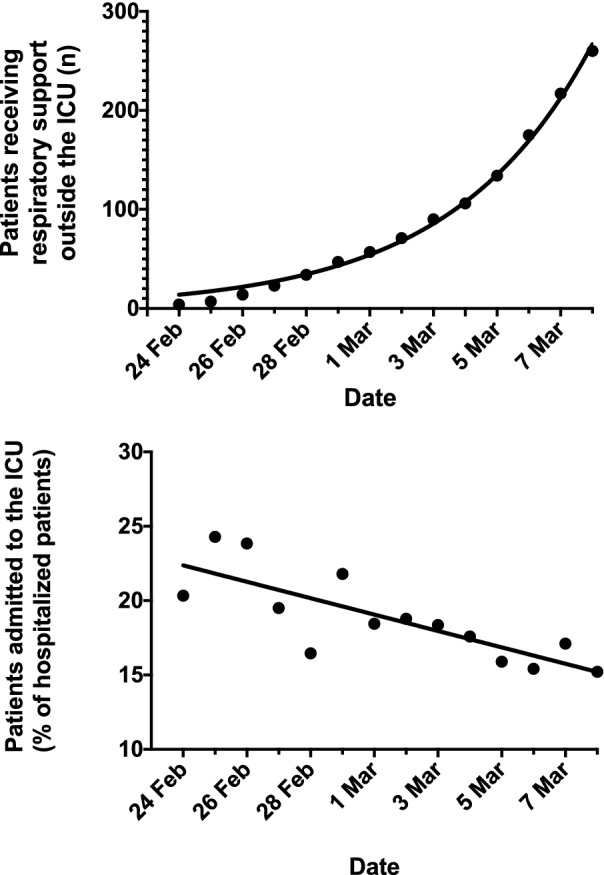
Number of patients assessed by the intensivist and treated outside the intensive care unit; fitted curve is exponential (top); proportions of patients admitted in the intensive care expressed as percentage of total hospitalized patients; linear fitting (bottom)

Data collection forms collected from the participating centers provided information on 542 patients treated in the ICU and on 260 patients who received respiratory support outside the ICU (802 patients in total). Notably, the number of patients receiving respiratory support outside the ICU increased from 4 (0.6%) on February 24 to 260 (37.0%) on March 8 (Fig. 1, top), and the proportion of patients admitted to the ICU declined from the 20.3% of hospitalized Covid-19 patients on February 24 to the 15.2% of hospitalized Covid-19 patients on March 8 (Fig. 1, bottom).

Compared to patients admitted to the ICU, patients receiving respiratory support outside the ICU were significantly older, had more cerebrovascular and renal comorbidities and fewer of them were obese. The attending intensivists deemed 189 patients (72.7% of the 260 patients treated outside the ICU) as non-eligible for further escalation of respiratory support (i.e., for invasive mechanical ventilation). In patients treated outside the ICU, conventional O_2_ therapy was applied in the 47.3% of the cases and non-invasive respiratory support (including NIV, CPAP and high-flow O_2_ therapy) in the 52.7%, while 81.8% of ICU patients were intubated. PaO_2_/FiO_2_ ratio, respiratory rate and arterial pH were higher and PaCO_2_ and base excess were lower in patients receiving respiratory support outside the ICU than in patients admitted to the ICU. (Table 1).

**Table 1:** Characteristics of patients receiving respiratory support in the ICU and outside the ICU under intensivist supervision

	Respiratory support		*p*-value
	In the ICU	Outside the ICU	
	*N* = 440	*N* = 260	
Age (median, IQR)	65 (56–73)	77 (65–82)	< 0.0001
Male (*n*, %)	352 (80.0)	188 (72.3)	0.048
Comorbidities (*n*, %)	*N = 433*	*N = 260*	
No comorbidities	73 (16.9)	0 (0.00)	< 0.0001
COPD	62 (14.3)	39 (15.0)	NS
Diabetes	103 (23.8)	50 (19.2)	NS
Obesity	136 (31.4)	40 (15.4)	< 0.0001
HIV infection	3 (0.7)	1 (0.4)	NS
Immunocompromised state	10 (2.3)	9 (3.5)	NS
Cardiovascular disease	275 (63.5)	158 (60.8)	NS
Cerebrovascular disease	25 (5.8)	34 (13.1)	< 0.001
Chronic kidney disease	23 (5.3)	26 (10.0)	0.0200
Chronic liver failure	6 (1.4)	3 (1.2)	NS
Active neoplasm	16 (3.7)	9 (3.5)	NS
No answer	100 (23.1)	43 (16.5)	0.0390
Respiratory support (*n*, %)	*N = 439*	*N = 241*	
Conventional O_2_ therapy	4 (0.9)	114 (47.3)	< 0.0001
HFNC	3 (0.7)	68 (28.2)	< 0.0001
Non-invasive respiratory support	69 (15.7)	59 (24.5)	0.0052
CPAP	28 (6.4)	18 (7.5)	NS
NIV	41 (9.3)	41 (17.0)	0.0033
Invasive mechanical ventilation	359 (81.8)	0 (0.0)	< 0.0001
Physiological variables^a^	*N = 425*	*N = 243*	
PaO_2_ pressure (mmHg)	92 (76–123)	62 (55–73)	< 0.0001
PaO_2_/FiO_2_ ratio	165 (117–230)	244 (147–296)	< 0.0001
PaCO_2_ (mmHg)	42 (37–50)	34 (31–38)	< 0.0001
pH	7.40 (7.34–7.45)	7.46 (7.43–7.49)	< 0.0001
Respiratory rate (breath/min)	20 (16–24)	24 (18–27)	< 0.0001
Base excess (mEq/L)	1.30 (− 1.10–3.90)	0.60 (− 2.5–2.0)	0.0018

*ICU* intensive care unit, *COPD* chronic obstructive pulmonary diseases, *HIV* human immunodeficiency virus, *HFNC* high-flow nasal cannula, *CPAP* continuous positive airway pressure, *NIV* non-invasive ventilation

^a^Worst values recorded during the first 24 h of admission

The infectious disease and the pneumology wards were the most more common locations where out-of-ICU respiratory support was delivered (47.2% and 30.9%, respectively) (Table 2). Patients receiving conventional O_2_ therapy outside the ICU had less cerebrovascular comorbidities and obesity and had significantly higher values of PaO_2_/FiO_2_ and arterial pH than patients receiving non-invasive ventilatory support outside the ICU (including NIV, CPAP and high-flow O_2_ therapy). Mortality did not differ between patients receiving conventional O_2_ therapy and non-invasive respiratory support (58.8% vs. 52.0%, respectively; Table 3).

**Table 2:** Reported allocations for administering ventilatory support outside the ICU

Allocation of patients (*n*, %)	*N* = 252
Intermediate care unit	17 (6.75)
Emergency medicine	38 (15.08)
Infectious disease ward	119 (47.22)
Pneumology ward	78 (30.95)

*ICU* intensive care unit

**Table 3:** Clinical and physiological variables of patients receiving respiratory support outside the ICU

	Conventional oxygen therapy (*N* = 114)	Non-invasive respiratory support^a^ (*N* = 127)	*p*-value
Age	80 (65–83)	75 (67–82)	NS
Male, *n* (%)	72 (63.2)	101 (79.5)	0.0048
Comorbidities (*n*, %)			
None	0 (0.0)	0 (0.0)	
COPD	18 (15.8)	21 (16.5)	NS
Diabetes	19 (16.7)	29 (22.8)	NS
Obesity	23 (20.9)	15 (11.8)	0.0570
HIV infection	0 (0.0)	0 (0.0)	
Immunocompromised state	4 (3.5)	2 (1.6)	NS
Cardiovascular disease	73 (64.0)	77 (60.6)	NS
Cerebrovascular disease	20 (17.5)	9 (7.1)	0.0127
Chronic kidney disease	13 (11.4)	11 (8.7)	NS
Chronic liver failure	2 (1.8)	1 (0.8)	NS
Active neoplasm	6 (5.3)	2 (1.6)	NS
Other	23 (20.2)	17 (13.4)	NS
Physiological variables^b^			
PaO_2_ pressure (mmHg)	65 (54–77)	59 (54–70)	0.0079
PaO_2_/FiO_2_ ratio	269 (198–323)	183 (102–265)	< 0.0001
PaCO_2_ (mmHg)	35 (31–38)	33 (30–38)	NS
pH	7.45 (7.41–7.48)	7.46 (7.44–7.50)	0.0234
Respiratory rate (breath/min)	24 (18–26)	24 (20–29)	NS
Base excess (mEq/L)	0.50 (− 2.53; 2.15)	0.85 (–0.85; 2.00)	NS
28-day mortality (*n*; %)	67 (58.8)	66 (52.0)	NS

^a^Non-invasive respiratory support includes non-invasive pressure support ventilation, continuous positive airway pressure, high-flow nasal cannula

^b^Worst values recorded during the first 24 h of admission

Analysis of 28-day mortality showed a proportion of deaths of 47.3% (260 out of 550) in patients treated in the ICU and of 52.1% (135 out of 259), in patients receiving respiratory support outside the ICU (*p* = 0.0112). Non-survivors treated in the ICU died within 11 (6–16) days while in non-survivors receiving respiratory support outside the ICU death occurred within 6 (4–11) days. Forty-four patients in the ICU group (8.0%) and 10 patients (3.9%) in the out-of-ICU group were still hospitalized through April 5th (last day of follow-up).

## Discussion

The present study describes how the Italian health-care system of three northern Italian regions responded to the increasing need for clinical resources for critically ill patients during the first 14 days of the Covid-19 outbreak through the 28.7% increase in ICU beds and the increasing use of non-invasive respiratory support outside the ICU.

Data to evaluate the impact of Covid-19 outbreak on the capacity of the health-care system to accomplish the need for ICU resources are limited. Xie and coworkers reported that in Wuhan as of Feb 10, 2020, there were about 1000 patients requiring ventilatory support with 120 new patients every day. However, since only 600 ICU beds were available, three general hospitals were rapidly converted to critical care hospitals with a total of about 2500 beds dedicated to Covid-19 critically ill patients [6]. Griffin and coworkers described the process to implement an ICU surge capacity at the greater New York Presbyterian system. In their experience, new COVID-19 ICUs had to be rapidly assembled after the first 3 weeks from the admission of the first critically ill Covid-19 patients [15].

Concomitantly to the increase in ICU bed capacity, there was a progressive increase in the number of patients who received respiratory support outside the ICU (from 0.6 to 37.0%) under the daily supervision of an intensivist. This allowed to reduce the percentage of patients admitted to the ICU from 20.3% on February 24th to 15.2% on March 8th. The response between the Italian and the greater New York Presbyterian systems was similar, despite the different ICU capacity (16.2% of the total hospital beds in the USA [16] vs. 2.8% in Italy (https://www.salute.gov.it/imgs/C_17_pubblicazioni_2859_allegato.pdf). This might be explained by the extensive use of out-of-ICU respiratory support we adopted in Italy [11–13].

Our data show that, compared to patients admitted to the ICU, patients receiving respiratory support outside the ICU were significantly older, had more comorbidities and had a higher PaO_2_/FiO_2_ ratio and a lower PaCO_2_. Among patients treated outside the ICU, proportions of patients treated with conventional O_2_ therapy and non-invasive respiratory support were comparable (47.3 vs. 52.7%, respectively). The median age of our ICU population [65 years (56–73)] is consistent with the one reported at national level in pre-pandemic times [17] and, although it is difficult to draw conclusion from these data, it is probable that the same age criteria were adopted during the first 2 weeks of the Covid-19 epidemics in Northern Italy.

Patients receiving conventional O_2_ therapy outside the ICU showed a PaO_2_/FiO_2_ ratio higher than those receiving non-invasive support outside the ICU, without differences in age and mortality. Although a crude comparison of mortality is not very informative because of the baseline differences between the ICU and out-of-ICU populations, we show here that the difference in survival at 28 days in patients treated in the ICU and those receiving respiratory support outside the ICU was small (47.3 vs. 52.1%, respectively). Altogether these data seem to suggest that treatment outside the ICU has been offered as a therapeutic setting proportional to patient’s conditions and not as a ‘limited’ standard of care, always remaining within the ethical perimeter of standard clinical practice [18, 19]. Nevertheless, is unlikely that all eligible patients were transferred to an ICU, and we cannot exclude that at least some patients who matched criteria for ICU admission did not survive long enough to be transferred to ICU or comorbid disease or goals of care precluded escalation to ICU level care.

Non-invasive ventilation was suggested to be avoided in Covid-19 patients due to the risk of transmission of infection [14]. In our hospitals, the risk might have been reduced for the following reasons: (a) helmets equipped with high-efficiency particulate air filters at the PEEP port were the interface of choice for delivering non-invasive respiratory support in almost 2/3 of patients treated outside the ICU; this interface might have avoided the dispersion of the multiphase turbulent gas cloud from coughing and sneezing on part of the patients, possibly reducing the transmission of COVID-19[20]; (b) about 50% of the patients receiving respiratory support outside the ICU were treated in infectious disease wards that are commonly equipped with negative pressure rooms [21]. Moreover, there is growing evidence that NIV can be safely performed outside the ICU in Covid-19 patients, and even advanced maneuvers such as prone positioning have been successfully tested in these patients [22].

These data have may important implications for the reorganization required by health-care systems necessary to manage the Covid-19 outbreak. The Italian Society of Anesthesia, Analgesia, Resuscitation, and Intensive Care (SIAARTI) recommended an approach for resource allocation based on “clinical appropriateness” and “distributive justice” in case of significant mismatch between the number of patients requiring ICU admission and the available resources and acknowledged that: “it is not about making choices on value, but to reserve possibly scarce resources first to who has higher probability of survival and second to who can have higher saved years of life, with the purpose of maximizing benefits for the highest possible number of people” [23].

Our data show that increasing the ICU capacity by 28.7% obtained through the reorganization of available facilities (conversion of operating rooms, coronary units, closure of all scheduled surgical activity) and use of out-of-ICU respiratory support [11–13], the health-care system was able to accomplish the clinical needs for respiratory support in Covid-19 patients and may suggest that end-of-life practices might have remained within the ethical perimeter of standard clinical practice [18, 19].

The retrospective nature represents the major weakness of this study. Although data have been collected by personnel with experience in clinical research and strongly motivated to share their experience, the enormous clinical load and the risk of contagion have certainly influenced the quality of the data and limited the number of information that has been possible to collect. Moreover, further analysis is needed to provide information regarding use of resources, allocation of beds, staffing choices, timing of opening up of new beds, and what resources were most stretched in the first 2 weeks. Moreover, the expected heterogeneity in hospital capacity and care practices between study hospitals may limit the practical utility of the description for clinicians facing an imminent surge of patients with COVID-19 disease. Despite these limitations, this study represents the first and most detailed description of the clinical reality of the first western country overwhelmed by the Covid-19 epidemic.

In conclusion, although our analysis confirms the grave concerns regarding the capacity of health-care systems to effectively respond to the Covid-19 outbreak, these data show that the rapid increase in beds obtained through the reversal of already available resources into intensive care facilities and the use of out-of-ICU respiratory support allowed to manage the first terrible 14 days of the Covid-19 outbreak. The present analysis shows that only rapid acquisition of new intensive care facilities with appropriate equipment and personnel and use of out-of-ICU respiratory support [11–13] may avoid the rationing of health-care resources that may be acceptable for “battlefield medicine”, but should be incompatible with health-care systems founded on the principles of universality, solidarity and distributive justice (article 32 of the Constitution of the Italian Republic and law number 833 December 23rd, 1978).

## Data Availability

The datasets used and/or analyzed during the current study are available from the corresponding author on reasonable request.
